# Association of mRNA expression of iron metabolism-associated genes and progression of non-alcoholic steatohepatitis in rats

**DOI:** 10.18632/oncotarget.25488

**Published:** 2018-05-25

**Authors:** Teruhisa Higuchi, Mitsuhiko Moriyama, Akiko Fukushima, Hiroshi Matsumura, Shunichi Matsuoka, Tatsuo Kanda, Masahiko Sugitani, Akiko Tsunemi, Takahiro Ueno, Noboru Fukuda

**Affiliations:** ^1^ Division of Gastroenterology and Hepatology, Department of Internal Medicine, Nihon University School of Medicine, Itabashi-Ku, Tokyo 173-8610, Japan; ^2^ Department of Pathology, Nihon University School of Medicine, Itabashi-Ku, Tokyo 173-8610, Japan; ^3^ Division of Nephrology, Hypertension and Endocrinology, Department of Internal Medicine, Nihon University School of Medicine, Itabashi-Ku, Tokyo 173-8610, Japan

**Keywords:** iron, NASH, SHRSP5 rat, intestine, HAMP

## Abstract

**Background:**

Excess iron is associated with non-alcoholic steatohepatitis (NASH).

**Results:**

mRNA expression of duodenal cytochrome b, divalent metal transporter 1, ferroportin 1, hepcidin, hephaestin and transferrin receptor 1 in liver were higher in high fat, high cholesterol-containing diet (HFCD) group than in normal diet (ND) group. mRNA levels of divalent metal transporter 1 and transferrin receptor 1, which stimulate iron absorption and excretion, were enhanced in small intestine. Epithelial mucosa of small intestine in HFCD group was characterized by plasma cell and eosinophil infiltration and increased vacuoles. Iron absorption was enhanced in this NASH model in the context of chronic inflammation of small intestinal epithelial cells, consequences of intestinal epithelial cell impairment caused by HFCD. Iron is transported to hepatocytes via portal blood, and abnormalities in iron absorption and excretion occur in small intestine from changes in iron transporter expression, which also occurs in NASH liver. Knockdown of hepcidin antimicrobial peptide led to enhanced heavy chain of ferritin expression in human hepatocytes, indicating association between hepcidin production and iron storage in hepatocytes.

**Conclusions:**

Iron-related transporters in liver and lower/upper portions of small intestine play critical roles in NASH development.

**Methods:**

Expression of iron metabolism-related genes in liver and small intestine was analyzed in stroke-prone spontaneously hypertensive rats (SHR-SP), which develop NASH. Five-week-old SHR-SP fed ND or HFCD were examined. mRNA and protein levels of iron metabolism-related genes in liver and small intestine from 12- and 19-week-old rats were evaluated by real-time RT-PCR and immunohistochemistry or Western blot.

## INTRODUCTION

Recently, the number of non-alcoholic steatohepatitis (NASH) cases and the number of cases with NASH-associated hepatocellular carcinoma (HCC) have increased [1–4]. However, the mechanism of disease progression remains unclear. It is considered that NASH is associated with various factors including lifestyle-associated issues such as obesity and diabetes mellitus (DM). In particular, it has been reported that these additional factors include genetic abnormalities, in addition to oxidative stress, high lipid peroxide levels, excess iron, insulin resistance and increased proinflammatory cytokine levels [5, 6].

The liver is supplied by portal blood flow from the intestine. It is reasonable to assume therefore that various substances from the intestine are involved in the pathology of liver diseases. Thus, the intestinal microenvironment also plays a role in the development of NASH and non-alcoholic fatty liver disease (NAFLD). The importance of the microbiome-gut-liver axis has long been recognized in human and rodent models [7–9]. Lipopolysaccharide (LPS), a product of gut bacteria, also influences the pathology of NASH [9–13].

In previous studies [14, 15], we investigated iron metabolism and liver pathology and reported that the progression of hepatitis C and the development of HCC are closely associated with iron metabolism. During the past few years, among the parameters evaluated as possible predictors of NAFLD, serum ferritin has emerged [16]. The expression levels of iron metabolism-related genes may affect the development of NASH. The environment of the small intestine, which is the site of iron absorption, may also influence iron metabolism.

Under normal conditions, mammalian cells acquire most of their iron from the plasma protein transferrin (Tf), which can deliver iron to cells and has a high affinity for binding to the plasma membrane protein Tf receptor 1 (TfR1) [17]. The main iron uptake transporter is the ferrous ion membrane transport protein divalent metal transporter 1 (DMT1) [17]. Solute carrier family 40 member 1 (SLC40A1)/ferroportin 1 (FPN1) essentially functions as an iron efflux transporter [17]. A ferric reductase that can deliver ferrous iron to DMT1 in the gut is duodenal cytochrome B (Dcytb), whose expression is enhanced by stimuli that enhance iron absorption [18]. Hepcidin is one of the regulators of iron release into plasma. In the small intestine, the copper-containing protein ceruloplasmin homolog hephaestin is required for efficient iron absorption, and animals with defective hephaestin function are anemic [18]. Cellular iron homeostasis is regulated by the iron transporters DMT1 and FPN1 and by the iron storage protein ferritin.

Spontaneously hypertensive rats (SHR) and stroke-prone spontaneously hypertensive rats (SHRSP) are well established parallel lines from outbred Wistar-Kyoto (WKY) rats [19, 20]. SHRSP5/Dmcr rats, a substrain of SHRSP, appear to be a useful model for analyzing the time-dependent changes of high fat, high cholesterol-containing diet (HFCD)-induced steatohepatitis and fibrosis progression [21, 22].

In the present study, we developed a NASH model using SHRSP5 rats fed HFCD and investigated the changes in mRNA expression of iron metabolism-related genes in the liver and the upper and lower parts of the small intestine. We found an association between the mRNA expression of iron metabolism-related genes and the progression of NASH. We also observed an association between the production of hepcidin and storage of iron in hepatocytes. We found iron-related transporters in the liver and in the lower and upper parts of the small intestine play critical roles in the development of NASH.

## RESULTS

### Comparisons of body, liver and spleen weights

The mean body weights of the ND and HFCD groups were 117.0 g and 114.5 g, respectively, at the beginning of the study, and at 7, 9, 11, 13, 15, 17 and 19 weeks of age were 198.1 g and 123.9 g, 237.3 g and 166.9 g, 282.3 g and 209.3 g, 289.9 g and 217.8 g, 307.0 g and 229.0 g, 294.3 g and 245.5 g and 336.8 g and 253.7 g, respectively. Body weight increase was less in the HFCD group than in the ND group. Of note, it was observed that all rats in the HFCD group frequently had diarrhea and became weaker over the course of the study.

Mean liver weights in the ND and HFCD groups at 12 and 19 weeks of age were 11.3 g and 23.5 g and 13.6 g and 34.6 g, respectively. Mean liver weight was significantly greater in the HFCD group than in the ND group at both 12 and 19 weeks of age (*p*<0.05 and *p*<0.05, respectively). Mean spleen weights in the ND and HFCD groups at 12 and 19 weeks of age were 0.53 g and 0.78 g and 0.5 g and 1.4 g respectively. Mean spleen weight was significantly greater in the HFCD group than in the ND group at both 12 and 19 weeks of age (*p*<0.05 and *p*<0.05, respectively).

### Iron content of liver and small intestine

We compared the amounts of iron in the liver and small intestine. The amount of iron in the livers of the HFCD group (9.58±0.94 ppb, n=4 at 12 weeks of age and 11.13±1.44 ppb, n=3 at 19 weeks of age) was significantly lower than that in the 5-week-old control group (20.2±5.25 ppb, n=4; *p*<0.05) and that in the ND group at 12 and 19 weeks of age (19.0±2.82 ppb, n=4; *p*<0.05 and 26.2±2.92 ppb, n=4; *p*<0.05, respectively). Similarly, the amount of iron in the small intestine in the HFCD group (7.25±0.070 ppb, n=4) was significantly lower than that in the ND group (8.45±0.35 ppb, n=4; *p*<0.05).

### Biochemical blood examination

Serum total bilirubin, γ-glutamyl transferase and free fatty acid levels at 19 weeks of age were significantly higher in the HFCD group than in the ND group. The serum total cholesterol level was significantly higher in the HFCD group than in the ND group at 12 weeks of age. Serum aspartate aminotransferase, alanine aminotransferase and alkaline phosphatase levels at both 12 and 19 weeks of age were significantly higher in the HFCD group than in the ND group (Table 1).

**Table 1:** Biochemical examination of blood samples obtained from normal diet (ND) and high fat and high cholesterol-containing diet (HFCD) groups at 5, 12 and 19 weeks of age

Parameters	week 5	week 12			week 19		
		ND (n=4)	HFCD (n=4)	*p-value*^a^	ND (n=4)	HFCD (n=3)	*p-value*^b^
AST (U/L)	251.75±19.97	167.50±8.96	587.00±167.40^c^	0.0012	276.75±82.43	976.00±203.07^c^	0.0001
ALT (U/L)	49.00±3.16	55.50±4.20	432.25±194.25^c^	0.0003	79.25±4.65	587.67±27.68^c^	0.0001
ALP (U/L)	1847.25±375.04	838.25±67.29^c^	2329.25±261.09^c^	0.0001	835.00±130.95^c^	2850.00±182.48^c^	0.0001
γ-GT (U/L)	2.90±0.0	2.90±0.0	2.90±0.0	N.S.	2.88±0.05	6.33±1.53^c^	0.0001
T.Bil (mg/dl)	0.06±0.01	0.06±0.01	0.05±0.01	N.S.	0.05±0.01	0.24±0.17^c^	0.0092
D.Bil (mg/dl)	0.03±0.01	0.02±0.01	0.02±0.01	N.S.	0.00±0.01	0.13±0.10^c^	0.0028
T.Chol (mg/dl)	86.75±7.68	56.00±1.41	226.75±28.40	0.00002	65.50±4.66	715.67±302.32^c^	0.0001
TG (mg/dl)	67.75±15.82	95.25±13.43	38.25±4.65	N.S.	84.25±22.34	90.00±76.39	N.S.
FFA (μEq/L)	512.50±113.81	499.75±143.19	591.25±73.09	N.S.	705.50±144.02	1267.00±325.29^c^	0.0046

AST, aspartate aminotransferase; ALT, alanine aminotransferase; ALP, alkaline phosphatase; γ-GT, γ-glutamyl transferase; T.Bil, total bilirubin; D.Bil, direct bilirubin; T.Chol, total cholesterol; TG, triglycerides; FFA, free fatty acids; Data are shown as mean ± SD. For each parameter, the Tukey-Kramer test was used for comparisons among the 5-week-old group and 12- and 19-week-old rats from the ND and HFCD groups. N.S., no statistically significant difference; ^a^ND versus HFCD groups; ^b^ND versus HFCD groups; ^c^p<0.05, compared with 5-week groups.

### Pathological findings in liver

In the ND group, liver fibrosis of the liver was not observed at either 12 or 19 weeks of age (Supplementary Figure 1A). Diffuse lipid droplets were seen in hepatocytes at both 12 and 19 weeks of age. However, perisinusoidal, pericellular and perivenular fibrosis, and ballooning of hepatocytes, which are typical indications of NASH, were not observed in the ND group. According to Brunt's criteria for NASH [23], activity grade 1, fibrosis stage 1 and activity grade 1, fibrosis stage 2 were observed in the 12- and 19-week-old ND groups, respectively. In addition, in the HFCD group at 12 weeks of age, greater numbers of lipid droplets in the livers were observed. Mild sinusoidal fibrosis, moderate pericellular fibrosis, and moderate perivenular fibrosis were also observed (Supplementary Figure 1A), as were ballooning hepatocytes and hyalines were also observed. These observations were consistent with NASH (activity grade 1, fibrosis stage 1) according to the Brunt's criteria [23].

**Figure 1:**
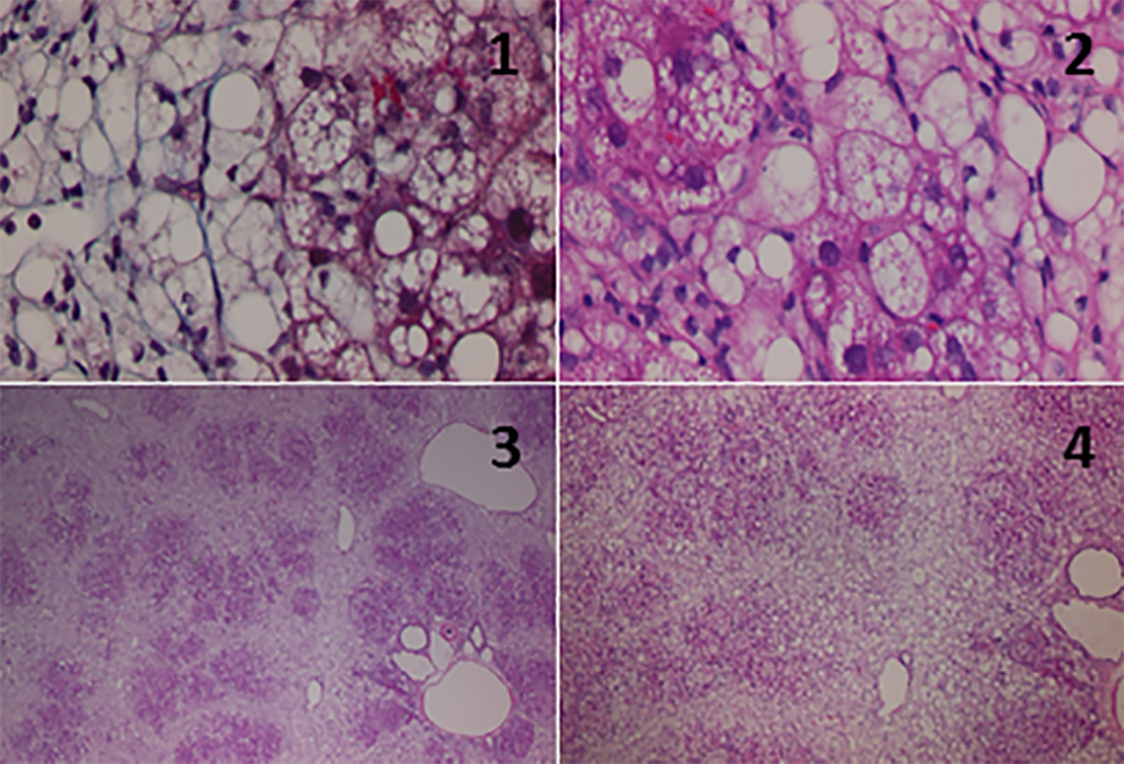
Histological findings for the livers from 19-week-old SHRSP5 rats from the high fat, high cholesterol-containing diet (HFCD) group Masson's trichrome stain **(1)** or hematoxylin and eosin (HE) stain **(2-4)**. Original magnification: x100 (1, 2) or x4 (3, 4). Ballooning hepatocytes and pericellular fibrosis were observed (1, 2). Enlargement of the portal vein and increased numbers of portal vein branches were observed (3). Massive necrosis (collapse) of hepatocytes was also observed (4).

Necro-inflammatory reaction was severe in the parenchymal area and mild in the periportal area. Inflammatory cell infiltration in the portal area was minimal. Moderate ballooning of hepatocytes was observed. In the HFCD group, the collapse of hepatocytes was observed at both 12 and 19 weeks of age. Fibrosis of the liver in the 19-week-old HFCD group indicated further progression. Moderate to advanced perisinusoidal fibrosis, pericellular fibrosis and perivenular fibrosis were observed. Furthermore, bridging fibrosis was also observed. These observations were consistent with NASH (activity grade 3, fibrosis stage 3) at 19 weeks of age based on the Brunt's criteria [23] (Figure 1).

Inflammatory cell infiltration was rather mild compared with that at 12 weeks of age, but there was more significant lipid droplet formation. Supplementary Figure 1B presents the characteristic changes in livers from the HFCD groups. As mentioned above, we considered that the livers from the 19-week-old HFCD group had histopathological features of NASH.

### Pathological findings of upper and lower portions of small intestine

It is assumed that the upper and lower portions of the small intestine are located near the duodenum and the ileum, respectively. In the upper segment, infiltration of inflammatory cells, including histiocytes, phagocytes and lymphocytes, was observed immediately below the mucosal epithelium in 5-week-old rats. In particular, infiltration of plasma cells was evident, and these histological features were consistent with chronic inflammation. There were a few lymphoid follicles. The nuclei of intestinal villus epithelial cells were arrayed along the bottom part of the cells, and the cell structure remained intact. The lower portion of the small intestine revealed almost the same findings as those from the upper portion at 5 weeks of age, although inflammatory cell infiltration was more apparent.

In the upper segment of the small intestine in the 19-week-old ND group, Auerbach's plexus was maintained, and infiltration of inflammatory cells, mostly plasma cells, was observed. There was chronic inflammation, but there was no difference in terms of severity compared to 5-week-old rats. In the lower segment of the small intestine in the 19-week-old ND group, there was marked detachment of the columnar epithelium, and deformation of the stroma was evident. We considered that these findings were caused by ischemia; however, necrosis in trophoblastic cells was not observed. Further, the number of vacuolated cells had also increased.

No columnar epithelial detachment was observed in the upper part of the small intestine in the 19-week-old HFCD group. The severity of inflammatory cell infiltration was similar to that in the ND group; infiltrating cells were mostly plasma cells, followed in number by lymphocytes and histiocytes. In addition, the number of cells with cytoplasmic vacuoles was greater in the 19-week-old HFCD group than in the ND group. These cytoplasmic vacuoles were not stained by Sudan III, meaning that they were not lipid droplets.

In the lower portion of the small intestine in the 19-week-old HFCD group, mild impairment of microvilli and the columnar epithelium was observed. The tissue structure was completely maintained, although moderate inflammatory cell infiltration in the stroma and signs of chronic inflammation were observed. In addition, cells with vacuoles in the cytoplasm were observed to the same degree as in the upper portion of the small intestine. Also, an increased number of goblet cells was observed compared with in the ND group.

### Expression of iron metabolism-related genes (IMGs) in the liver

**Figure 2:**
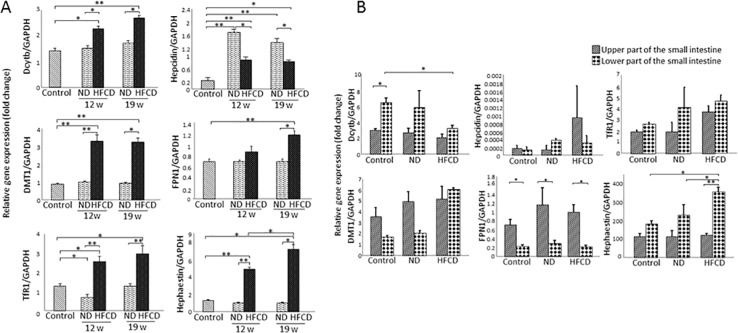
Real-time RT-qPCR analysis of the expression of iron metabolism-related genes (IMGs) in the liver and small intestine of SHRSP5 rats from the high fat, high cholesterol-containing diet (HFCD) or normal diet (ND) groups **(A)** Expression of IMGs in the liver. **(B)** Expression of IMGs in the small intestine. Data represent mean ± standard deviation. For each parameter, the Turkey-Kramer test was used for comparisons among the 5-week-old group and the 12- and 19-week-old rats from the ND and HFCD groups. (^*^P<0.05; ^**^P<0.001). FPN1, ferroportin 1; TfR1, transferrin receptor 1; DMT1, divalent metal transporter 1; Dcytb, duodenal cytochrome b; GAPDH, glyceraldehyde-3-phosphate dehydrogenase.

IMG mRNA expression in the liver was examined at 5 weeks, 12 weeks and 19 weeks of age (Figure 2A). Of the IMGs, Dcytb, DMT1, TfR1, hepcidin, FPN1 and hephaestin mRNA expression increased significantly at 12 and 19 weeks of age in the HFCD group compared with the control group. Further, hephaestin mRNA levels at 19 weeks of age increased significantly compared to those at 12 weeks of age in the HFCD group. Conversely, hepcidin mRNA levels in the HFCD group were significantly higher in the 5-week-old control group but were significantly lower than those in the ND group (at both age 12 and 19 weeks). Moreover, hephaestin is the only gene with significantly increased mRNA levels between age 12 and 19 weeks.

Hepcidin is the only gene whose mRNA level increased more than the levels of the 5-week-old ND group. A significant difference in mRNA expression levels between the ND and HFCD groups was observed for Dcytb, DMT1, TfR 1, FPN1 and hephaestin. In addition, hepcidin was the only gene with significantly lower mRNA expression in the HFCD group than in the ND group.

### IMG mRNA expression in small intestine

Dcytb was the only gene with enhanced mRNA expression in the lower segment compared to the upper segment of the small intestine in the control subjects, whereas FPN1 was the only gene with enhanced mRNA expression in the upper segment compared to the lower segment in the control subjects (Figure 2B).

In the upper portion of the small intestine, Dcytb was the only gene that demonstrated a tendency to decrease compared to 5-week-old rats. On the other hand, DMT1, hepcidin and TfR1 showed a tendency to increase compared to 5-week-old rats (Figure 2B). In the lower portion of the small intestine, DMT1, TfR1 and hephaestin demonstrated a tendency to increase in comparison to 5-week-old rats (Figure 2B).

### Western blot analysis of iron metabolism-related proteins

**Figure 3:**
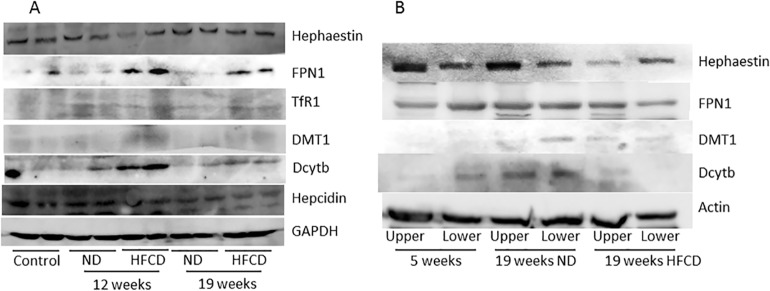
Western blot analysis of iron metabolism-related proteins in the liver **(A)** and upper and lower portions of the small intestine **(B)** of SHRSP5 rats from the high fat, high cholesterol-containing diet (HFCD) or normal diet (ND) groups. FPN1, ferroportin 1; TfR1, transferrin receptor 1; DMT1, divalent metal transporter 1; Dcytb, duodenal cytochrome b; GAPDH, glyceraldehyde-3-phosphate dehydrogenase.

Compared with the 5-week-old group, Dcytb, DMT1, TfR1 and FPN1 expression were higher, and hepcidin expression was lower in the 19-week-old HFCD group, while hephaestin expression was not changed (Figure 3A).

In the upper portion of the small intestine, Dcytb, DMT1 and FPN1 expression levels were higher in the HFCD group than in the 5-week-old group, whereas hephaestin expression was lower in the HFCD group than in the 5-week-old group. In the lower portion of the small intestine, DMT1 expression was enhanced in the HFCD group, but Dcytb and FPN1 expression levels were lower than those in the 5-week-old group (Figure 3B).

### Immunohistochemical analysis (IHC) of iron metabolism-related molecules at protein level

Next, we performed immunohistochemical (IHC) analysis to confirm the results from Western blot analyses, (Supplementary Figures 2 and 3). Comparisons of the degree of expression of the various proteins in the liver and in the upper and lower segments of the small intestine are shown in Supplementary Figures 2 and 3. At the protein level in the liver, the expression levels of Dcytb, DMT1 and FPN1 were higher in the HFCD group at 12 and 19 weeks of age than those in the 5-week-old group (Supplementary Figure 2A-2D). The expression of hepcidin was higher in the HFCD group at age 12 and 19 weeks than that at age 5 weeks. In addition, there was a marked increase in hepcidin expression in the cytoplasm as well as in the cell membrane of hepatocytes in the HFCD group. The hephaestin level in hepatocytes was greater in the HFCD group than in the ND group at both ages. In sinusoidal cells, increased DMT1, FPN1, hephaestin and hepcidin expression levels were observed in the HFCD group compared to the ND group, but differences were not statistically significant.

In the upper portion of the small intestine, Dcytb and hephaestin expression in the HFCD group tended to be lower, and DMT1 and FPN1 expression tended to be higher in the HFCD group than in the 5-week-old group. No differences in Dcytb, DMT1, FPN1 or hephaestin expression were observed in the ND group compared with the 5-week-old group (Supplementary Figure 3A-3D).

In the lower portion of the small intestine, DMT1, FPN1 and hephaestin expression tended to be higher, and the expression of Dcytb was lower in the HFCD group than in the 5-week-old group. No differences were observed in Dcytb, FPN1, and hephaestin expression between the ND and the 5-week-old groups (Supplementary Figure 3A to 3D).

### Knockdown of hepcidin antimicrobial peptide (HAMP) induced heavy chain of ferritin (FTH) mRNA expression in human hepatoma cell lines

**Figure 4:**
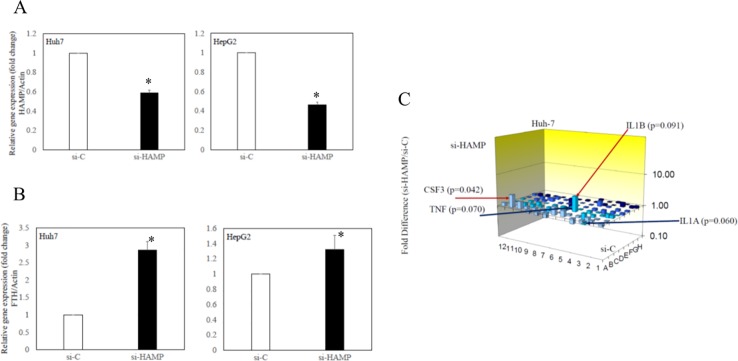
Effects of knockdown of hepcidin antimicrobial peptide (HAMP) on ferritin and toll-like receptor (TLR)-associated gene expression in human hepatoma cell lines **(A)** HAMP mRNA expression and **(B)** heavy chain of ferritin (FTH) mRNA expression were examined by RT-qPCR. **(C)** Changes of TLR-associated gene expression in Huh7 cells transfected with si-HAMP (siRNA against HAMP) or si-C (control siRNA). Data represent mean ± standard deviation. ^*^P<0.05, compared with si-C-transfected cell lines.

To confirm the association between the production of hepcidin and the storage of iron in hepatocytes, ferritin mRNA expression was examined in human hepatoma cell lines transfected with si-HAMP compared with those transfected with si-C (Figure 4A-4B). These siRNAs were validated by real-time PCR (Figure 4A). A differential regulation of the two ferritin subunits FTL and FTH has been reported [24], and also that dietary iron supplementation affected human FTH [25]. The knockdown of HAMP induced FTH expression in human hepatoma cell lines (Figure 4B).

TLR-associated signaling target PCR array was also performed using Huh7 transfected with si-HAMP and those transfected with si-C. IL1β and colony stimulating factor 3 (CSF3) were upregulated 3.0-fold and 2.5-fold in Huh7 transfected with si-HAMP, compared with si-C-transfected cells (p=0.091 and p=0.042, respectively). TNF and IL1α were downregulated 2.3-fold and 2.2-fold in Huh7 transfected with si-HAMP (p = 0.070 and p = 0.060, respectively) (Figure 4C). The expression of HAMP mRNA might be associated with cytokine gene expression in human hepatocytes.

## DISCUSSION

In livers of the 19-week-old group, it is suggested that the increased expression of DMT1 and FPN1, which are iron-related transporters, in the HFCD group enhanced the absorption and excretion of iron (Figure 2A). In addition, hepcidin expression, which is negatively regulated by promotion of the degradation of FPN1, was increased in the cytoplasm and cell membrane of hepatocytes as well as in mesenchymal cells, compared with the 5-week-old group. On this basis, the iron excretion mechanism was activated in the hepatocytes of the HFCD group. Furthermore, it is suggested that an increase in the reduction to Fe^2+^, as well as the oxidization of excreted Fe^2+^, was positively regulated by increased Dcytb and hephaestin expression (Figure 2A).

Expression of the iron reductase gene Dcytb in the lower portion of the small intestine of the 19-week-old group was less in the HFCD group than in the 5-week-old group, and Dcytb expression also tended to be less in the upper portion of the small intestine (Figure 2B). On the other hand, a high level of FPN1 expression was observed in only the upper portion of the small intestine, both DMT1 and TfR1 expression levels were higher in both the upper and lower portions, and hephaestin expression was higher in only the lower portion in the HFCD group (Figure 2B). Based on these results, it was confirmed that the uptake and excretion of iron were increased in the upper portion of the small intestine in the HFCD group. Additionally, in the lower portion of the small intestine, it was confirmed that Fe^2+^ uptake was increased and oxidation of excreted Fe^2+^ was increased in the HFCD group. As mentioned above, iron absorption and iron excretion were stimulated in intestinal epithelial cells of the HFCD group by increased expression of DMT1 and FPN1, as well as the increased expression of TfR1, which is an iron receptor.

Dcytb expression in the upper part of the small intestine tended to be lower in the HFCD group (Figure 2B); therefore, it was confirmed that the reduction from Fe^3+^ to Fe^2+^ is suppressed, which makes the uptake of Fe^2+^ into intestinal epithelial cells difficult. In addition, it is assumed that the oxidation of Fe^2+^ excreted from the basal membrane side of intestinal epithelial cells was not stimulated because there was no change in hephaestin expression.

On the other hand, the results from the upper segment of the small intestine differed slightly from those of the lower segment. In the lower segmentof the small intestine of the HFCD group, both Dcytb and FPN1 expression levels decreased over time; while DMT1 expression increased, as did hephaestin expression (Figure 2B). Therefore, we confirmed that the reduction of Fe^3+^ is regulated in an inhibitory manner, but the absorption of iron is regulated in a stimulatory manner in the lower part of the small intestine; in addition, the oxidation of Fe^2+^ excreted from the basal membrane side of intestinal epithelial cells acted in a stimulatory manner. On this basis, it is assumed that the host transfers Fe^2+^, which binds iron as a complex to solid organs such as the liver.

Expression of iron metabolism-related proteins tended to be increased in intestinal epithelial cells that were histologically damaged or degenerated. We observed that the absorption and excretion of iron were stimulated in epithelial cells of the upper segment of the small intestine and that the absorption of iron was stimulated in epithelial cells of the lower segment of the small intestine, but excretion was suppressed in the HFCD group. We found that the reduction of Fe^3+^ was stimulated. The reduction of Fe^3+^ was inhibited by decreased Dcytb (Figure 3B), although our data regarding protein expression were less compelling, perhaps in part because of the technical difficulties encountered in obtaining strictly comparable tissue samples.

On the other hand, in studies on IMGs in rats that develop NASH, hepcidin expression has been reported to be decreased in the liver [26] and FPN1 expression is stimulated [27]. Furthermore, it has been reported that liver diseases other than NASH may be associated with DMT1 gene polymorphisms [28] and the upregulation of Dcytb expression [29]. As mentioned above, it is assumed that iron metabolism is more complex in patients with hepatic diseases. However, there are few studies on iron absorption pathways in the small intestine [30].

Of note, hephaestin mRNA expression was markedly increased in the liver and lower small intestine. Therefore, we suppose that hephaestin has a novel activity in the onset of NASH. Furthermore, goblet cells are significantly increased in the upper and lower small intestine. We inferred that the small intestine, whose primary function is absorption, protects the mucosa by increasing mucous secretory cells, such as goblet cells, in response to exposure to substances such as HFCD.

One mechanism for the development of NASH involves LPS, a TLR4 agonist derived from enterobacterial flora; LPS reaches the liver in large amounts and increases the secretion of proinflammatory cytokines, such as tumor necrosis factor (TNF)-alpha, by stimulating Kupffer cells [10, 13, 31]. The proportion of Lactobacilli in feces was increased in a study of the distribution of enteric bacteria in feces in an experimental NASH model created by feeding HFCD [12]. As mentioned above, complicated mechanisms are involved in the development of NASH, and they are mediated by interactions between the intestinal tract and the liver. These mechanisms include iron metabolism. Therefore, we considered that studies on the morphology of the small intestine and IMGs are valuable for confirming the involvement of the enterohepatic relationship in the development of NASH. We believed that exposure of the small intestinal mucosa to HFCD would cause intestinal epithelial cell damage, leading to a breakdown of the absorption and excretion pathways of iron, mediated by the oxidation and reduction of iron and iron transporters and causing the development of NASH.

Regarding the morphology of the small intestine in the HFCD group, we considered that there was severe impairment of the epithelium of the small intestinal mucosa, likely through exfoliation and necrosis, as the rats in the present study displayed marked weakness caused by frequent diarrhea and weight loss. Considerable impairment of the epithelial cells was not observed, although degeneration of the small intestinal mucosa and mild exfoliation occurred. Moreover, a marked increase in the infiltration of plasma cells and an increase in eosinophil infiltration in the small intestine were observed.

As mentioned above, we considered that feeding HFCD induced inflammatory cell infiltration in the epithelium of the small intestine and increased the permeability of the mesentery and, as a result, induced abnormalities in the absorption and excretion of various metabolites such as LPS and minerals including iron. Consequently, abnormalities in the expression of iron oxidoreductase and iron transporters in small intestinal epithelial cells were induced and excess iron was transported to hepatocytes and mesenchymal cells via the portal blood flow. In addition, we assumed that abnormalities in the absorption and excretion of iron caused by abnormal expression of the receptor genes and oxidation-reduction-related genes in hepatocytes also contributed to the development of NASH.

Next, we examined the expression of IMGs in the liver. The weights of the livers were significantly greater in the HFCD group than in the ND group; however, the total amount of iron in the livers was significantly lower in the HFCD group than in the ND group. This NASH model may lead to severe impairment and degeneration of hepatocytes, which store iron intrinsically, as well as a reduced number of Kupffer cells, as lipid droplets were diffusely increased in hepatocytes. These findings are supported by the facts that increases in the production of hepcidin and FPN1 by hepatocytes would induce iron excretion and that knockdown of HAMP induced FTH mRNA expression (Figure 4B). The present study also demonstrated an association between altered expression of IMGs in the liver of rats and the development of NASH.

Using Western blot analysis, we observed that, although the expression level of hepcidin mRNA increased, the amount of hepcidin protein in the hepatocytes decreased slightly. The exact reason for this finding remains unclear. Unfortunately, measuring and interpreting the iron content is difficult because a large amount of fat accumulates in rat livers in this NASH model. The expression level of hepcidin tended to be lower at 19 weeks of age compared with that at 12 weeks of age in the HFCD group. Our protein expression data are less compelling, perhaps in part because of the technical difficulties encountered in obtaining strictly comparable tissue samples.

When we examined intrahepatic iron (Fe^3+^) deposition by Prussian blue staining, we were unable to find any iron deposition in the liver in this NASH rat model. Thus, we confirmed that the presence of Fe^3+^ in the liver was extremely low in this NASH rat model. We are planning to confirm the amount of iron (Fe^2+^ and Fe^3+^) in the liver in future studies by applying other methods, including iron staining.

Additionally, IMG expression was examined in the small intestine. When the upper and lower segments of the small intestine were compared, differences in the expression of genes and proteins were noted. In epithelial cells in the upper segment, there was no significant change in Dcytb expression in the HFCD group, regardless of HFCD feeding. However, marked promotion of the uptake of Fe^2+^ uptake into intestinal epithelial cells due to an increase in DMT1 expression and a significant increase in TfR1 expression was seen.

On the other hand, FPN1 expression tended to be higher in the upper segment of the small intestine, and the excretion of Fe^2+^ was stimulated, whereas there was no change in hephaestin expression. Therefore, it is suggested that regulation exists to prevent the oxidization of excreted Fe^2+^ to Fe^3+^ through FPN1 in epithelial cells in the upper portion of the small intestine.

In epithelial cells in the lower portion of the small intestine, suppression of the reduction of Fe^3+^ to Fe^2+^ caused by decreased Dcytb expression, increased DMT1 expression, and increased uptake of Fe^2+^ caused by increased TfR1expression was seen in the HFCD group. This pattern of expression was clearly different from that in the liver. On the other hand, no change was seen in hepcidin expression, while FPN1 expression decreased and hephaestin expression increased in the lower portion of the small intestine, resulting in excreted Fe^2+^ being oxidized to Fe^3+^ in epithelial cells of the upper small intestine. As mentioned above, feeding HFCD caused a decrease in Dcytb expression and increases in both DMT1 and hephaestin expression.

Under normal conditions, iron is absorbed from the gastrointestinal content of the upper segment of the small intestine. However, increases in DMT1 and TfR1 expression were also observed in the lower segment in the HFCD group. In addition, there was no change in hepcidin expression although an increase in hephaestin expression was seen. Therefore, it can be assumed that feeding HFCD increased the uptake of iron in the lower portion of the small intestine, enhanced the excretion of iron from intestinal cells to the portal circulation, and enhanced the oxidation of excreted Fe^2+^ to Fe^3+^. Based on these results, we considered that iron absorption and excretion are regulated in the small intestine and liver as follows. First, prolonged intake of HFCD disrupts the intestinal flora, causing abnormal enteric bacteria to propagate. It is thought that the host reacts to prevent the propagation of abnormal enteric bacteria by actively absorbing iron into intestinal epithelial cells to reduce the level of iron that is required for their growth. Then, the host promotes iron absorption by overexpressing the genes required for the excretion of iron so that the absorbed iron is excreted into the portal blood flow. Next, to protect mucosal cells from damage from HFCD, mucus secretory cells, such as goblet cells, proliferate, and the small intestine, which is primarily an absorption organ, secretes considerable mucus to protect the small intestinal mucosa.

On the other hand, this is regulated so that the excretion of iron and the expression of genes that oxidize excreted Fe^2+^ to Fe^3+^ are increased. As mentioned above, it is assumed that the expression of iron metabolism-related proteins, which are involved in the absorption, transport and excretion of iron, is involved in the development of NASH in a complex manner. In particular, it is assumed that changes in enterobacterial flora caused by HFCD influence the pathophysiological state. Further studies on the relationship between the intestinal tract and the liver in the development of NASH will be needed.

Iron absorption was stimulated in this NASH model as a consequence of the impairment of intestinal epithelial cells caused by HFCD. Excess iron is transported to hepatocytes via the portal blood and abnormalities in iron absorption and excretion, caused by the altered expression of iron transporters, also occur in the hepatocytes of this NASH model. There is an association between the mRNA expression of iron metabolism-related genes and the progression of NASH. There is also an association between the production of hepcidin and the storage of iron in hepatocytes. Iron-related transporters in the liver and in both lower and upper segments of the small intestine play critical roles in the development of NASH.

In conclusion, iron absorption was stimulated in this NASH model as a consequence of the impairment of intestinal epithelial cells caused by HFCD. Excess iron is transported to hepatocytes through portal blood, and abnormalities in iron absorption and excretion, caused by the altered expression of the iron transporters, also occur in the hepatocytes of this NASH model.

## MATERIALS AND METHODS

### SHRSP5 rats

The arteriolipidosis-prone rat (ALR; SHRSP5) used in the present study is a subline obtained by feeding HFCD to SHRSP [19, 20]. These rats are characterized by fat deposition in their arteries and fibrosis of the liver, leading to the development of diet-induced NASH. When SHRSP5 rats were fed HFCD, blood biochemical and liver histological findings were comparable to those of rats fed a stroke-prone (SP) diet at weeks 8 and 14 [20]. Based on these experiments, we created a NASH rat model by feeding HFCD, which consisted of 68% (w/w) SP diet (Funabashi Farm, Chiba, Japan), 25% (w/w) palm oil, 5% (w/w) cholesterol and 2% (w/w) cholic acid, to ALRs in accordance with the experimental protocol previously described [21, 22].

The normal diet (ND) group was fed only the SP diet. These rats arrived at our laboratory at 4 weeks old and were used for the experiments after being fed ND for 1 week in our laboratory. This investigation conformed to the Guide for the Care and Use of Laboratory Animals published by the US National Institutes of Health (NIH Publication No. 85-23, 1996). The Ethics Committee of Nihon University School of Medicine examined all research protocols involving the use of animals and approved this study.

### Biochemical blood examination

See Supplementary Material.

### Histological examination

Initially, 4 SHRSP5 rats each from the HFCD and ND groups were sacrificed by exsanguination under anesthesia using sodium pentobarbital (Nembutal, 50 mg/kg IP; DS Pharma Biomedical, Osaka, Japan) at week 7 (12 weeks of age) or week 14 (19 weeks of age). The controls were four 5-week-old SHRSP5 rats. Blood was collected from the inferior vena cava, and serum was immediately isolated and stored at −80°C. Livers and the upper and lower segments of the small intestines were collected and divided into three portions. First, the tissues were fixed in 2.5% formalin, embedded in paraffin, and stored at 4°C. Second, the tissues were embedded in optimal cutting temperature (OCT) compound immediately after collection, rapidly frozen in a dry ice/acetone bath, and stored at −80°C until use. Finally, the remaining tissues were stored at −80°C without fixation for future use. Hematoxylin and eosin (HE) staining and Masson's trichrome staining were performed for histological examination of liver tissue. In addition, the upper and lower portions of the small intestine were observed using only HE staining (see Supplementary Material for details).

### Examination of iron metabolism-related genes

mRNA expression levels of iron metabolism-related genes (IMGs), including Dcytb, DMT1, TfR1, FPN1 hepcidin, and hephaestin, were measured by real-time reverse transcription-quantitative polymerase chain reaction (RT-qPCR). The mRNA expression of IMGs in the liver and upper and lower segments of the small intestine was studied and compared among the 5-week-old group and the ND and HFCD groups. Primers used in the present study are shown in Supplementary Table 1 (see Supplementary Material for details).

### Western blot analysis

Western blot analysis was performed to determine the levels of various proteins in the organs. Immunoblotting of Dcytb, DMT1, TfR1, FPN1, hephaestin, hepcidin and DMT1 was performed as previously described by Arakawa et al [32] (see Supplementary Material for details).

### Immunohistochemical analysis

Immunohistochemical (IHC) staining for proteins encoded by iron metabolism-related genes was performed using paraffin-embedded sections of liver tissue obtained from 5-, 12- and 19-week-old rats and paraffin-embedded sections of the upper and lower portions of the small intestine obtained from 5- and 19-week-old rats. Indirect IHC staining was performed for the detection of Dcytb, DMT1, FPN1, hepcidin, and hephaestin in the liver and the upper and lower portions of the small intestine (see Supplementary Material for details).

### Cell culture, transfection of siRNAs, gene expression analysis and human toll-like receptor (TLR)-associated signaling target PCR array

Human hepatoma cell lines Huh7 and HepG2 were grown in Roswell Park Memorial Institute medium (RPMI-1640) (Sigma-Aldrich, St. Louis, MO, USA) supplemented with 10% fetal bovine serum (FBS) at 5% CO2 and 37°C [33, 34]. The siRNA against HAMP (si-HAMP) and control siRNA (si-C) were obtained from Santa Cruz Biotechnology (Santa Cruz, CA, USA). Transfection of 50 nM siRNAs was performed with Effectene Transfection Reagents (Qiagen, Hilden, Germany) as previously described [34]. After 48 hours, extraction of cellular RNA, cDNA synthesis and RT-qPCR were performed based on the SYBR-Green methods, using a 7500 Fast real-time PCR system (Applied Biosystems, Foster, CA, USA) as previously described [34]. PCR amplification was performed on cDNA templates using primers specific for human actin (sense primer 5′-CAGCCATGTACGTTGCTATCCAGG-3′ and antisense primer 5′-AGGTCCAGACGCAGGATGGCATG-3′), HAMP (sense primer 5′-TGCCCATGTTCCAGAGGC-3′ and antisense primer 5′-CCGCAGCAGAAAATGCAGAT-3′) and FTH (sense primer 5′-AATTGGGTGACCACGTGACC-3′ and antisense primer 5′-TTCCGCCAAGCCAGATTC-3′) [35]. Gene expression analysis was performed using the ΔΔCT (comparative cycle threshold) method [34]. We also performed a human TLR-associated signaling target PCR array according to the manufacturer's protocol [34]. The data were analyzed using PCR Array Data Analysis Software (http://www.sabiosciences.com/pcrarraydataanalysis.php).

### Statistical analysis

The study included 20 rats, but one was then excluded. The amount of mRNA detected and the results of biochemical examination of blood are presented as mean ± standard deviation (SD). Statistical analyses were performed using the Tukey-Kramer test for multiple comparisons. Staining scores of tissues were analyzed using the Steel-Dwass test for multiple comparisons. A statistically significant difference was defined as *P* < 0.05. All statistical analyses were performed using JMP 12 (SAS Institute Inc., Cary, NC, USA).

## SUPPLEMENTARY MATERIALS FIGURES AND TABLES



## Abbreviations

ALR: SHRSP5 rat (arteriolipidosis-prone rat)
ND: normal diet
HFCD: high fat and high cholesterol-containing diet
IMGs: iron metabolism-related genes
Dcytb: duodenal cytochrome b
DMT1: divalent metal transporter 1
TfR1: transferrin receptor 1
FPN1: ferroportin 1
HE: hematoxylin and eosin
IHC: immunohistochemistry
mRNA: messenger RNA
NAFLD: non-alcoholic fatty liver disease
NASH: non-alcoholic steatohepatitis
RT-qPCR: real-time reverse transcription-quantitative polymerase chain reaction
LPS: lipopolysaccharide
HAMP: hepcidin antimicrobial peptide
si-C: control siRNA
si-HAMP: siRNA against HAMP.
