# Supplementary material for: Association of mRNA expression of iron metabolism-associated genes and progression of non-alcoholic steatohepatitis in rats
Source: Oncotarget. 2018 May 25;9(40):26183–94. doi: 10.18632/oncotarget.25488 (PMC5995254; doi:10.18632/oncotarget.25488)
Supplement: Supplementary file 1 [file oncotarget-09-26183-s001.pdf]

Association of mRNA expression of iron metabolism-associated genes and progression of non-alcoholic steatohepatitis in rats

SUPPLEMENTARY MATERIALS

Biochemical blood examinations

For biochemical examination of blood, the levels of total bilirubin (T.Bil), direct bilirubin (D.Bil), aspartate aminotransferase (AST), alanine aminotransferase (ALT), alkaline phosphatase (ALP), γ-glutamyl transferase (γ-GT), total cholesterol (T.Cho), triglycerides (TG) and free fatty acids (FFA) were measured immediately after blood collection. T.Bil and D.Bil levels were measured by the chemical oxidation method. AST, ALT, ALP and γ-GT were measured by methods corresponding to the JSCC standard. The T.Cho level was measured by the cholesterol dehydrogenase (UV) method, the TG level by an enzymatic method (GK-GPO, free glycerol elimination method), and the FFA level by an enzymatic method.

Examination of iron metabolism-related genes

RNA was extracted from specimens that had been kept at -80ºC, and complementary DNA (cDNA) was prepared using conventional methods. RT-qPCR was performed using the Step One Plus™ Real Time PCR System (Applied Biosystems, Norwalk, CT, USA) with the SyBr Green-based-ddCt methods. The PCR primer sequences used for detection are shown in Supplementary Table 1.

Western blot analysis

The total membrane fraction was isolated using RIPA buffer [Nacalai, Kyoto, Japan, 50 mM Tris-HCl (pH.7.6), 150 mM NaCl, 1% Nonident P140, 0.5% sodium deoxycholate, plus protease inhibitor cocktail] containing 1% Triton X-100 (Roche Applied Science, Penzberg, Germany). After transfer, the membrane was blocked in 5% skim milk and incubated with anti-Dcytb antibody (ab66048 rabbit polyclonal antibody, Abcam, Cambridge, UK), anti-TfR1 antibody (SAB4200398 rabbit polyclonal antibody, Sigma-Aldrich, St. Louis, MO, USA), anti-SLC40A1(FPN1) antibody (ab85370 rabbit polyclonal antibody, Abcam), anti-actin antibody (ab3280 mouse monoclonal antibody, Abcam), anti-hephaestin antibody (sc-49969 goat polyclonal antibody, Santa Cruz Biotechnology, Dallas, TX, USA), anti-hepcidin antibody (ab81010 rabbit polyclonal antibody, Abcam) or anti-DMT1 antibody (ab140977 rabbit polyclonal antibody, Abcam) for 1 hour. After washing 3 times with 0.02 M Tris-buffered saline containing 0.1% Tween 20 (Sigma-Aldrich), the membrane was incubated with horseradish peroxidase-conjugated anti-goat IgG antibody (Bio-Rad Laboratories, Hercules, CA, USA) or anti-mouse IgG antibody (Zymed, San Francisco, CA, USA) for 1 hour. Bands were visualized using a chemiluminescence method (ECL plus Western blotting detection system, GE Healthcare, Buckinghamshire, UK) with the luminescent image analyzer LAS-3000 (Fuji Film, Tokyo, Japan).

Immunohistochemical analysis

Anti-Dcytb antibody (Abcam, ab66048 rabbit polyclonal Ab), DMT1 antibody (Abcam, ab140977 rabbit polyclonal Ab), anti-SLC40A1 (FPN1) antibody (Abcam, ab85370 rabbit polyclonal Ab), anti-hepcidin antibody (including hepcidin 25; Abcam, ab81010 rabbit polyclonal Ab), and anti-hephaestin antibody (Santa Cruz Biotechnology, sc-49969 goat polyclonal Ab) were used as primary antibodies. IHC staining for hepcidin in the small intestine was not performed because its expression level was assumed to be low.

Liver tissue sections were deparaffinized by the usual method, followed by antigen activation using target retrieval solution (DAKO, 1699). Endogenous peroxidase was removed by 3% hydrogen peroxide plus methanol and endogenous antigens were blocked using a blocking solution containing normal serum from an animal immunized with secondary antibody. Primary antibodies were used after 100-fold dilution and were incubated with tissue sections at room temperature for 30 minutes and then at 4ºC overnight. For secondary antibodies, Nichirei histofine simple stain rat (Nichirei, Histofine Simple Stain™, rat), MAX-PO (MULTI) (Nichirei code 414171, Tokyo, Japan) and MAX-PO (G) (Nichirei code 414331) were used and incubated with tissue sections at 37ºC for 1 hour. Color was developed using Nichirei simple stain DAB solution (Nichirei code 415171), followed by eosin staining and water rinsing, and was observed after mounting. Regarding localization in the liver, the cytoplasm and nuclei of cells were examined. The periportal, parenchymal, and portal area, and sinusoidal cells were evaluated region by region. The degree of localization was scored and compared using a five-grade evaluation system (0: none [no positive cells]; 1: minimal [several positive cells in lobules]; 2: mild [several positive cells in each lobule]; 3: moderate [half of cells in each lobule are positive]; 4: marked [positive cells are diffusely present in all lobules]). Each section of the upper and lower portions of the small intestine was scored and compared using a five-grade evaluation system (0: none

[no positive cells]; 1: minimal [several positive cells in the entire tissue]; 2: mild [several positive cells in each villus]; 3: moderate [half of the cells in each villus are positive]; 4: marked [positive cells are diffusely present in all villi]).

Measurement of iron contents

Tissues were pretreated as follows. About 50 mg of frozen tissue sample was placed into a polypropylene centrifuge tube and 5 ml of 0.1 mol/L nitric acid (high purity) was added. The tissue sample was suspended by stirring. These samples were kept at 2-8ºC for 72 hours. Tubes were centrifuged at 4,000 rpm at 5ºC for 10 minutes. 500 µl of the resultant supernatant was added to 4,500 µl of 0.1 mol/L nitric acid (high purity) to make 10-fold dilutions, and the iron content in these sample solutions was measured using an Inductively Coupled Plasma Mass Spectrometry-Atomic emission spectrophotometer (ICP-AES, Shimazu ICP8100, Kyoto, Japan).

A

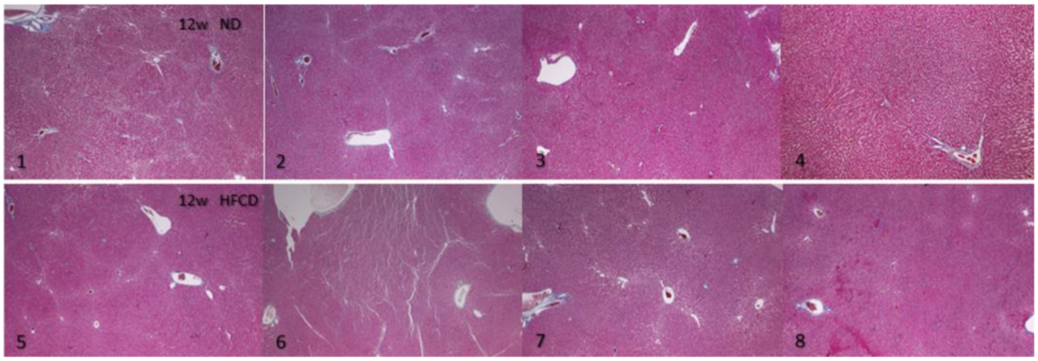

B

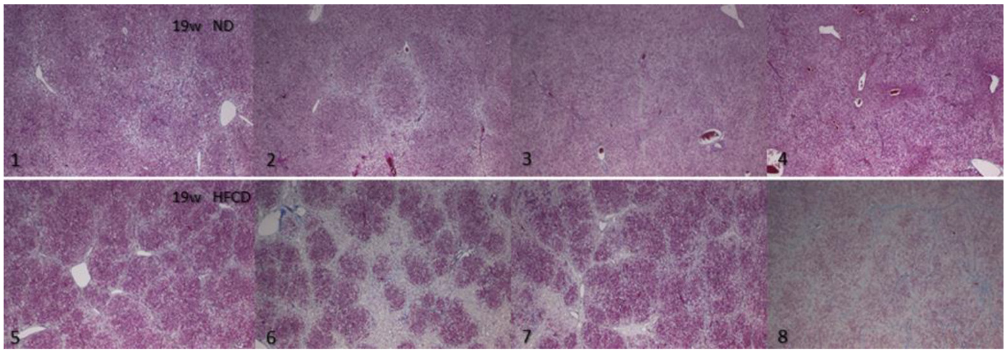

Supplementary Figure 1: Histological findings of the liver and small intestine of 12-week-old and 19-week-old SHRSP5 rats from high fat and high cholesterol-containing diet (HFCD) and normal diet (ND) groups. (A) Representative images showed the ND group (1-4) and HFCD group (5-8) at 12 weeks old. Brunt's classification [23] in the ND and HFCD groups was NASH (activity grade 1, fibrosis stage 1) and NASH (activity grade 1, fibrosis stage 1), respectively. Hematoxylin and eosin (H.E.) stain. Original magnification: x4. **(B)** Representative images showed the ND group (1-4) and HFCD group (5-8) at 19 weeks old. Brunt's classification [23] in the ND and HFCD groups was NASH (activity grade 1, fibrosis stage 2) and NASH (activity grade 3, fibrosis stage 3), respectively. Masson’s trichrome stain. Original magnification: x4.

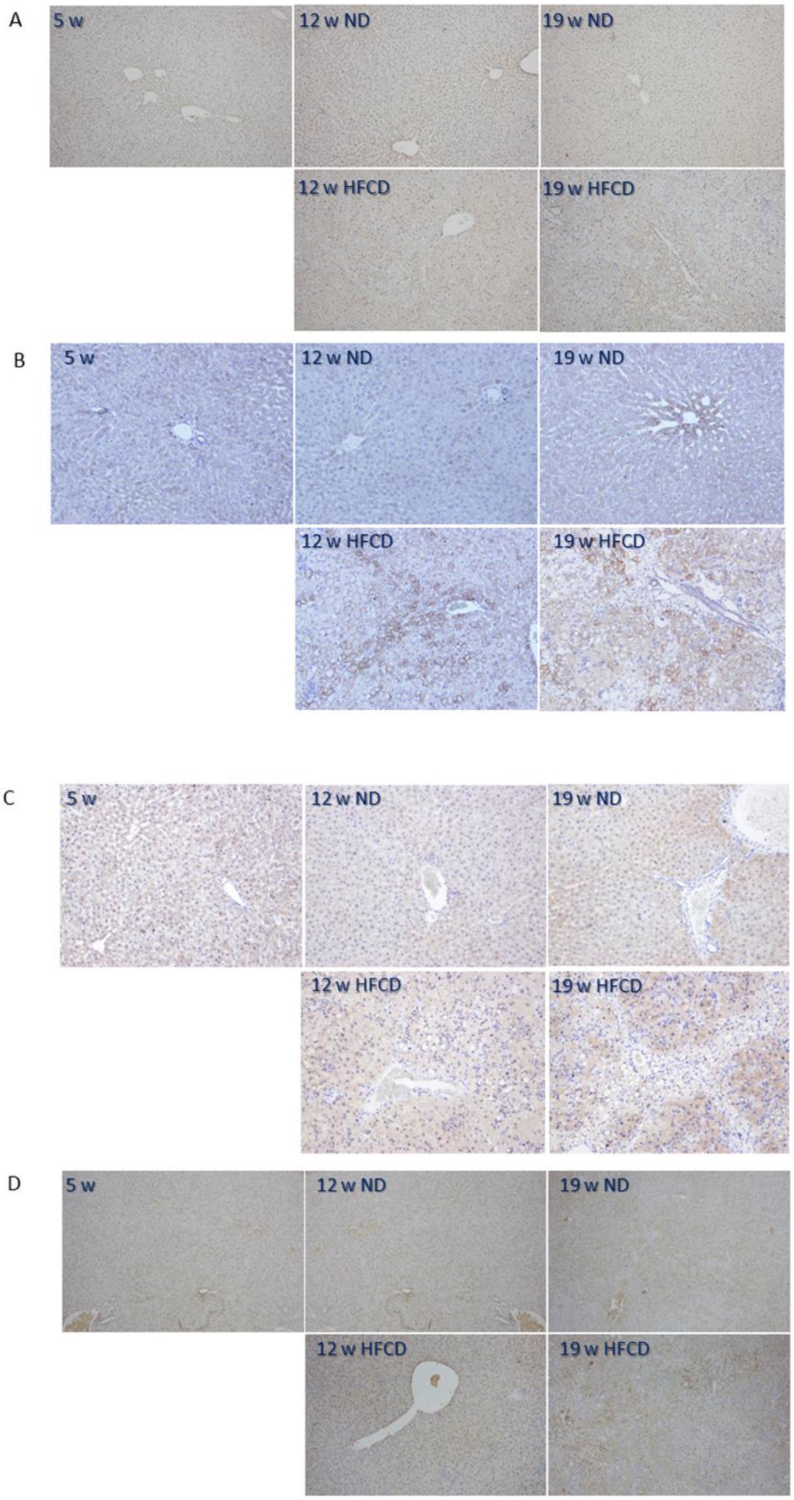

Supplementary Figure 2: Localization of iron metabolism-related proteins in liver at 5 weeks, 12 weeks and 19 weeks old in ND and HFCD groups detected by immunohistochemistry (IHC). **(A)** Duodenal cytochrome b (Dcytb). Dcytb expression in the cytoplasm and sinusoidal cells was higher than that in the five-week-old group. Dcytb expression was higher in the HFCD group than in the ND group. **(B)** Divalent metal transporter 1 (DMT1). DMT1 expression in the cytoplasm and sinusoidal cells in the 19-week-old group was higher than in the 5-week-old group. DMT1 expression was higher in the HFCD group than in the ND group. **(C)** Ferroportin 1 (FPN1). In the 12- and 19-week-old groups, FPN1 expression in the cytoplasm and sinusoidal cells was higher than in the five-week-old group. FPN1 expression was higher in the HFCD group than in the ND group. **(D)** Hepcidin. In the 12- and 19-week-old groups, hepcidin expression was higher than in the five-week-old group. Hepcidin expression was higher in the HFCD group than in the ND group.

(*Continued*)

E

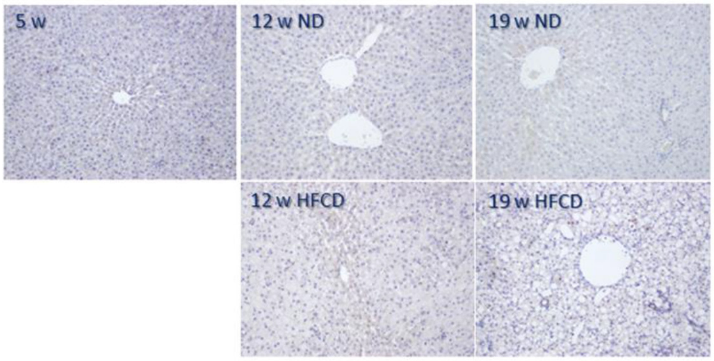

Supplementary Figure 2 (*Continued*): (E) Hephaestin. No significant difference in hephaestin expression was observed between the 12- and 19-week-old groups, but the expression was higher in sinusoidal cells. Original magnification: x10.

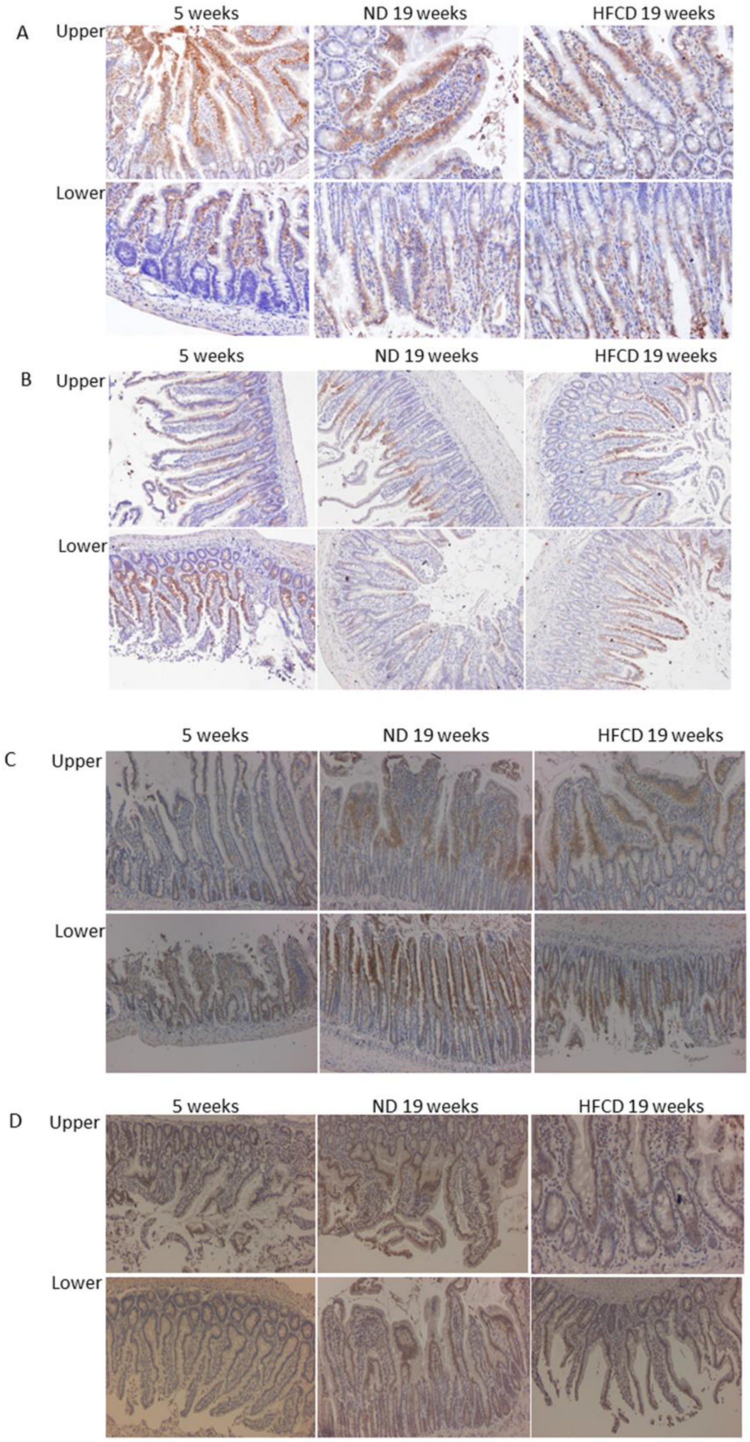

Supplementary Figure 3: Localization of iron metabolism-related proteins in upper and lower portions of the small intestine at 5 weeks and 19 weeks old in ND and HFD groups by IHC. **(A)** Duodenal cytochrome b (Dcytb). In the upper portion of the small intestine, no difference was observed between the HFD group and 5-week-old group. In the lower portion of the small intestine, Dcytb expression was lower in the HFD group than in the 5-week-old group. **(B)** Divalent metal transporter 1 (DMT1). In both the upper and lower portions of the small intestine, the expression was higher in the HFD group than in the 5-week-old group. In the lower portion of the small intestine, the expression in the ND group was higher than in the 5-week-old group. **(C)** Ferroportin 1 (FPN1). In the upper portion of the small intestine, the expression was higher in the HFD group than in the 5-week-old group. In the lower portion, the expression was lower in the HFD group than in the 5-week-old group. **(D)** Hephaestin. In the upper portion of the small intestine, the expression was lower in the HFD group than in the 5-week-old group. In the lower portion of the small intestine, the expression was higher in the HFD group than in the 5-week-old group. Original magnification: x10.

Supplementary Table 1: Primers used for real-time quantitative PCR

Genes	Forward/Reverse primers	Sequences
Dcytb	Forward	5´-TTAGCCATTGCCAACAAGCC-3´
	Reverse	5´-GGCTGTGAGTATCCTCGTCCAA-3´
Tfr1	Forward	5´-AGTGGTCGCTGGGTGTGATT-3´
	Reverse	5´-CCTTCAGGCATACAGCTCAATTG-3´
DMT1	Forward	5´-ATAGCAGCAGCCCCCATG-3´
	Reverse	5´-AGGCCCGAAGTAACATCCAA-3´
Hephaestin	Forward	5´-GGCACAGTTACAGGGCAGATG-3´
	Reverse	5´-ACATGGTCAGTAACGTGGCAGT-3´
Hepcidin	Forward	5´-TGACAGTGCGCTGCTGATG-3´
	Reverse	5´-GGAATTCTTACAGCATTTACAGCAGA-3´
FPN1	Forward	5´-GGTGGTGGCAGGCTCTGT-3´
	Reverse	5´-TTTGAACCACCAGGGACGTC-3´
GAPDH	Forward	5´-CAACTCCCTCAAGATTGTCAGCAA-3´
	Reverse	5´-GGCATGGACTGTGGTCATGA-3´

Dcytb, duodenal cytochrome b; Tfr1, transferrin receptor 1; DMT1, divalent metal transporter 1; FPN1, ferroportin 1; GAPDH, glyceraldehyde-3-phosphate dehydrogenase.
